# A nomogram for predicting recurrence in endometrial cancer patients: a population-based analysis

**DOI:** 10.3389/fendo.2023.1156169

**Published:** 2023-11-07

**Authors:** Mengdan Miao, Yanping Zhu, Lulu Wang, Yifei Miao, Rong Li, Huaijun Zhou

**Affiliations:** ^1^ Affiliated Drum Tower Hospital, Medical School, Nanjing University, Nanjing, China; ^2^ Department of Gynecology, Nanjing Drum Tower Hospital Clinical College of Nanjing Medical University, Nanjing, China

**Keywords:** endometrial cancer, recurrence, risk factor, nomogram, predictive model

## Abstract

**Objective:**

Endometrial cancer recurrence is one of the main factors leading to increased mortality, and there is a lack of predictive models. Our study aimed to establish a nomogram predictive model to predict recurrence in endometrial cancer patients.

**Method:**

Screen 517 endometrial cancer patients who came to Nanjing Drum Tower Hospital from 2008 to 2018. All these data are listed as the training group, and then 70% and 60% are randomly divided into verification groups 1 and 2. Univariate, Multivariate logistic regression, stepwise regression were used to select variables for nomogram. Nomogram identification and calibration were evaluated by concordance index (c-index), area under receiver operating characteristic curve (AUC) over time and calibration plot Function. By decision curve analysis (DCA), net reclassification index (NRI), integrated discrimination improvement (IDI), we compared and quantified the net benefit of nomogram and ESMO-ESGO-ESTRO model-based prediction of tumor recurrence.

**Results:**

A nomogram predictive model of endometrial cancer recurrence was established with the eight variables screened. The c-index (for the training cohort and for the validation cohort) and the time-dependent AUC showed good discriminative power of the nomogram. Calibration plots showed good agreement between nomogram predictions and actual observations in both the training and validation sets.

**Conclusions:**

We developed and validated a predictive model of endometrial cancer recurrence to assist clinicians in assessing recurrence in endometrial cancer patients.

## Introduction

As an epithelial cancer, endometrial cancer (EC) forms in the endometrium and is prevalent in perimenopausal and menopausal women. Current research indicates that EC is the most prevalent gynecological cancer in affluent nations (1–3). Moreover, its incidence rose from 2013 to 2017 (4, 5). According to the most current worldwide cancer statistics published in 2020 by the IARC of WHO, EC is second among prevalent genital tract malignancy after cervical cancer (6). In recent years, due to lifestyle changes and the popularity of hormone replacement therapy, the incidence of EC has gradually increased and gradually become younger (7).The progression of EC is relatively slow, and it is often detected at an early stage (8), so the prognosis is relatively good (9). However, there are still about 20% of patients with recurrence and metastasis (10), resulting in increased mortality (11, 12).

Age, grade and type of histology, myometrial invasion, and lymphovascular space invasion (LVSI) represent the risk factors for conventional endometrial cancer recurrence (12) and based on these risk variables, a set of recurrence patterns have been created. Some instances are EC recurrence patterns based on the European Society for Medical Oncology (ESMO), the European Society for Radiotherapy & Oncology (ESTRO), and the European Society of Gynaecological Oncology (ESGO) (ESMO-ESGO-ESTRO) consensus conference (8, 13, 14). However, these methods’ limitations are their relatively low accuracy and inferior capacity for the prediction of recurrence risk among individuals. Thus, tailoring a model for predicting EC patients is necessary.

The nomogram tumor prediction model has been extensively utilized recently (15–17). For instance, the nomogram prediction model was used to construct treatment and monitoring regimens for patients in stages IIIB and IIIC in melanoma (18) and hepatocellular carcinoma to predict the recurrence of patients after laparoscopic liver resection (19). The nomogram tumor prediction model meets the requirements of an ensemble model and plays a role in promoting personalized medicine, valuable to physicians for recurrence prediction (15). In this study, we used 517 endometrial cancer patients who visited Nanjing Drum Tower Hospital from 2008 to 2018 to establish a nomogram tumor recurrence prediction model for EC.

## Materials and methods

### Study population

The only participants in this retrospective cohort research were endometrial cancer patients who attended Nanjing Drum Tower Hospital between 2008 and 2018. The following are the requirements for inclusion: (1) patients diagnosed with endometrial cancer based on clinical manifestations, auxiliary examinations, and postoperative pathology; and (2) case records including age, menopausal status, clinical stage, histological tumor grade, radiotherapy history, chemotherapy history, preoperative CA125, preoperative ultrasound results, postoperative CA125, reproductive history, histological type, cervical infiltration, vascular infiltration, metastasis, and surgical approach. The exclusion criteria included patients who were not regularly followed up.

### Cohort partitioning and variable filtering

All patient data were used for the training group, and 60% and 70% were randomly selected as validation group 1 and validation group 2. For the first cohort, it intended to filter the factors for model production. Meanwhile, validation sets verify the former group’s outcomes. The collected data included 15 variables: age, menopausal status, clinical stage, histological tumor grade, radiotherapy history, chemotherapy history, preoperative CA125, preoperative ultrasound results, postoperative CA125, reproductive history, histological type, cervical invasion, vascular invasion, metastasis, and surgical approach. Among them, the diagnostic criteria of CA125 in Nanjing Drum Tower hospital was the normal range of 0-30.2U/ml, when the serum CA125 level >30.2U/ml, it was defined as an elevated CA125 level. Surgical approaches were divided into non-surgical treatments based on radiotherapy and chemotherapy, minimally invasive surgery (laparoscopic/Da Vinci robotic), or open surgery. Univariate logistic regression was performed on all 15 variables; those part of stepwise regression and multivariate logistic regression analyses were those having p < 0.1. In latter analysis, those having p < 0.05 were deemed independent risk factors, and regression analysis selected variables for the nomogram based on the Akaike information criterion (AIC). Finally, a total of eight variables were screened out.

### Statistical analysis

The percentage of missing data was 4.20%, and the missing ratio of each item was less than 20%. Missing data was performed with multiple imputations using IBM SPSS 26 with complete conditional specification (MCMC). The model type for scale variables was predicted mean matching (PMM), and all variables were used as predictors. To evaluate the recurrence probability of EC, factors were included in the nomogram using univariate logistic regression, multivariate logistic regression, and stepwise regression based on the minimal value of the AIC. The capacity to recognize was examined using the consistency index (C-index)/receiver operating characteristic curve (ROC), as well as related ability to calibrate was evaluated with the calibration chart. Related values have a 0.5-1.0 range denoting random to perfect probability. In general, those values > 0.7 imply acceptable estimations. For assessing the nomogram’s effectiveness with respect to the ESMO-ESGO-ESTRO pattern, integrated discrimination improvement (IDI), net reclassification index (NRI), and decision curve analysis (DCA) were utilized. NRI and IDI examine prediction advancements involving risks as well as novel models’ utility (20, 21); the other assesses predictive models’ viability (22, 23) through calculating the net benefit at various threshold likelihoods on the nomogram.

We evaluated age distributions across the training and validation groups using one-way ANOVA, and other clinicopathological parameters, such as clinical stage, were compared using cross-tab chi-square tests. Two-sided P values were considered, with values < 0.05 deemed to have significance statistically. For all statistical studies, R v.4.0.2 or SPSS 26 were used. The main endpoint of the trial was relapse, as measured by the period between diagnosis and all-cause recurrence or the date of the final follow-up in 2018. The nomogram risk was divided into low, medium, media-high, and high risk using the 0.25, 0.5, and 0.75 cutoffs, using the model predicted value. Risk stratification based on ESMO-ESGO-ESTRO was utilized for categorizing patients as low-risk, intermediate-risk, intermediate-high-risk, or high-risk. The Kaplan-Meier curve of recurrence-free survival (RFS) was developed for assessing accuracy in prediction on each approach.

## Results

### Patients’ characteristics

Between 2008 and 2018, a total of 671 patients were diagnosed with endometrial cancer at Nanjing Drum Tower Hospital, of whom 517 were qualified for the research. Patients lost to follow-up were excluded. All 517 patients were applied to the training cohort to construct a predictive mode. Of these patients, 70% (364) and 60% (312) were randomly selected for the validation cohort. Comparable clinical features existed across the training and validation groups (P>0.05) according to **Table 1**, which summarizes the clinical features of these EC patients.

**Table 1:** Clinical demographics of EC patients.

Characteristic	Whole population [cases (%)]	Validation cohort1 [cases (%)]	Validation cohort2 [cases (%)]	P value
**Total**	517	364	312	
Age				
Median	58	58	58	0.943
Mean	57.29	57.46	57.56	
Clinical stage				
I	398 (77.0)	286 (78.6)	244 (78.2)	0.998
II	41 (7.9)	27 (7.4)	22 (7.1)	
III	55 (10.6)	36 (9.9)	32 (10.3)	
IV	23 (4.4)	15 (4.1)	14 (4.5)	
Menopause status				
Pre	91 (17.6)	63 (17.3)	58 (18.6)	0.979
Peri	87 (16.8)	63 (17.3)	49 (15.7)	
Post	339 (65.6)	238 (65.4)	205 (65.7)	
Histologic grade				
Low grade	175 (33.8)	121 (33.2)	102 (32.7)	0.699
Media grade	246 (47.6)	171 (47.0)	139 (44.6)	
High grade	96 (18.6)	72 (19.8)	71 (22.8)	
Radiation therapy				
No	273 (52.8)	195 (53.6)	170 (54.5)	0.894
Yes	244 (47.2)	169 (46.4)	142 (45.5)	
Chemotherapy				
No	292 (56.5)	204 (56.0)	174 (55.8)	0.979
Yes	225 (43.5)	160 (44.0)	138 (44.2)	
Preoperative CA125				
Negative	384 (74.3)	275 (75.5)	224 (71.8)	0.532
Positive	133 (25.7)	89 (24.5)	88 (28.2)	
Positive ultrasound				
Negative	40 (7.7)	29 (8.0)	26 (8.3)	0.954
Positive	477 (92.3)	335 (92.0)	286(91.7)	
Postoperative CA125				
Negative	482 (93.2)	338 (92.9)	295 (94.6)	0.648
Positive	35 (6.8)	26 (7.1)	17 (5.4)	
Reproductive history				
Yes	469 (90.7)	332 (91.2)	285 (91.3)	0.944
No	48 (9.3)	32 (8.8)	27 (8.7)	
Histological type				
Endometrioid adenocarcinoma	470 (90.9)	333 (91.5)	281 (90.1)	0.815
Others	47 (9.1)	31 (8.5)	31 (9.9)	
Cervical invasion				
Negative	498 (96.3)	351 (96.4)	298 (95.5)	0.794
Positive	19 (3.7)	13 (3.6)	14 (4.5)	
Vascular invasion				
Negative	430 (83.2)	295 (81.0)	249 (79.8)	0.450
Positive	87 (16.8)	69 (19.0)	63 (20.2)	
Metastasis				
Negative	475 (91.9)	335 (92.0)	287 (92.0)	0.996
Positive	42 (8.1)	29 (8.0)	25 (8.0)	
Surgical approach				
No surgery	21 (4.1)	12 (3.3)	11 (3.5)	0.967
Minimally invasive	340 (65.8)	239 (65.7)	202 (64.7)	
Open	156 (30.2)	113 (31.0)	99 (31.7)	
Recurrence				
No	455 (88.0)	321 (88.2)	275 (88.1)	0.996
Yes	62 (12.0)	43 (11.8)	37 (11.9)	

Pre (<6 months since last menstrual period (LMP) AND no prior bilateral ovariectomy AND not on estrogen replacement),Peri (6-12 months since LMP),Post (>12 months since LMP or prior bilateral ovariectomy).

In the training cohort, validation cohort 1 and validation cohort 2, the median EC patient age in years was 58. Relapsed patients accounted for 12.0%; 8.1% of patients had metastases upon diagnosis; 4.1% of patients received radiotherapy or chemotherapy without surgery; 30.2% received laparotomy; 65.8% of patients underwent minimally invasive laparoscopic or da Vinci robotic surgery.

### Nomogram variable screening

In multivariate logistic regression analysis, only those having P < 0.1 in univariate regression analysis had been included, including age, clinical stage, histological grade, radiotherapy history, chemotherapy history, preoperative CA125, preoperative ultrasound results, postoperative CA125, pathological type, cervical invasion, vascular invasion, and metastasis. In multivariate logistic regression analysis, age, clinical stage, CA125 levels after surgery and surgical technique were identified as independent predictive variables for EC. The findings of stepwise regression showed that, within the training cohort, the model, including age, chemotherapy history, preoperative ultrasound results, postoperative CA125, cervical invasion, vascular invasion, and surgical approach, had the smallest AIC value, with an AIC value of 303.03. Therefore, we included eight factors, including age, clinical stage, history of chemotherapy, positive ultrasound, postoperative CA125, cervical invasion, vascular invasion, and surgical approach, in constructing the nomogram prediction model for EC recurrence (**Table 2**).

**Table 2:** Univariate and multivariate logistic analyses on variables for the prediction of recurrence of EC patients.

	Univariate logistic analysis			Multivariate logistic analysis		
Variable	HR	95% CI	P value	HR	95% CI	P value
**Age**	1.05	1.03-1.08	**<0.001**	1.06	1.03-1.09	**<0.001**
Clinical stage						
I	1.00			1.00		
II	2.62	1.00-6.14	**0.035**	2	0.67-5.38	0.2
III	5.22	2.57-10.38	**<0.001**	2.11	0.66-6.56	0.188
IV	9.79	3.88-24.25	**<0.001**	4.09	1.19-13.81	**0.023**
Menopause_status						
Pre	1.00					
Peri	0.8	0.27-2.24	0.668			
Post	1.43	0.70-3.23	0.353			
Histologic grade						
Low grade	1.00			1.00		
Media grade	1.15	0.59-2.31	0.680	1.09	0.48-2.53	0.839
High grade	3.36	1.67-6.94	**0.001**	1.83	0.76-4.47	0.179
Radiation therapy						
No	1.00			1.00		
Yes	0.35	0.19-0.62	**0.001**	0.71	0.31-1.62	0.422
Chemotherapy						
No	1.00			1.00		
Yes	2.88	1.66-5.11	**<0.001**	1.54	0.68-3.57	0.306
Preoperative CA125						
Negative	1.00			1.00		
Positive	2.35	1.35-4.05	**0.002**	1.14	0.56-2.25	0.706
Positive ultrasound						
Negative	1.00			1.00		
Positive	0.17	0.01-0.83	**0.088**	0.3	0.02-1.63	0.258
Postoperative CA125						
Negative	1.00			1.00		
Positive	6.94	3.29-14.43	**<0.001**	5.82	2.27-14.95	**<0.001**
Reproductive history						
Yes	1.00					
No	0.78	0.35-1.97	0.563			
Histological type						
Endometrioid adenocarcinoma	1.00			1.00		
Others	0.35	0.17-0.74	**0.004**	1.06	0.43-2.81	0.897
Cervical invasion						
Negative	1.00			1.00		
Positive	5.98	2.23-15.43	**<0.001**	2.3	0.59-9.08	0.228
Vascular invasion						
Negative	1.00			1.00		
Positive	3.3	1.82-5.87	**<0.001**	1.86	0.87-3.92	0.105
Metastasis						
Negative	1.00			1.00		
Positive	2.54	1.13-5.31	**0.017**	0.49	0.15-1.51	0.221
Surgical approach						
No surgery	1.00			1.00		
Minimally invasive	0.16	0.06-0.50	**0.001**	0.11	0.03-0.44	**0.001**
Open	0.72	0.27-2.15	0.533	0.42	0.12-1.54	0.174

P values that have statistical significance (less than 0.05) were in bold. Confidence interval is denoted by CI, while hazard ratio is represented by HR.

### Prediction of recurrence in women with endometrial cancer

**Figure 1:**
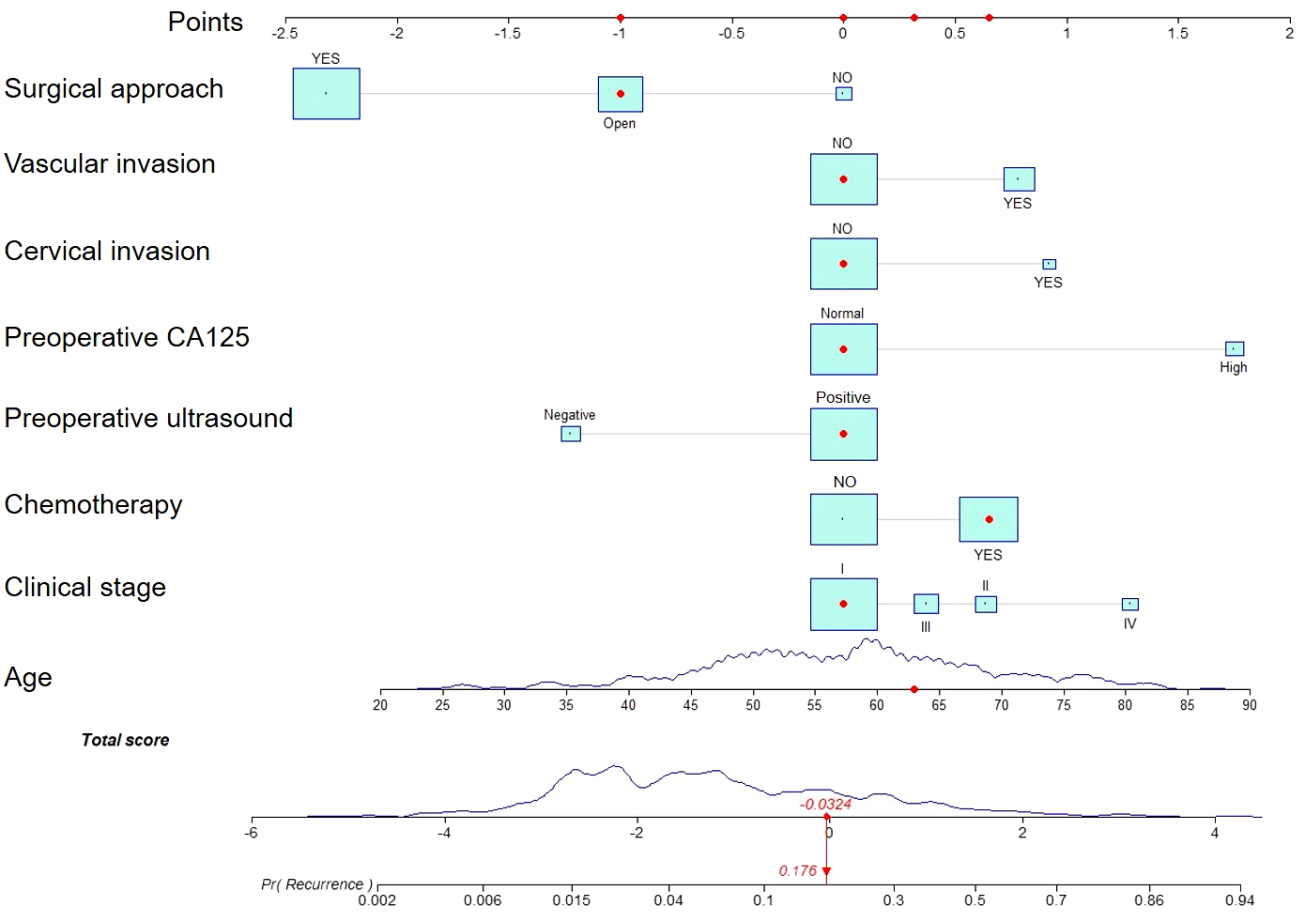
A nomogram for predicting endometrial cancer recurrence in patients. Estimating risk requires drawing a line from the patient’s variable value to the “Points” axis and counting the number of points for each variable. To establish the recurrence likelihood for this patient, the total score was computed by summing the points of all factors, and a straight line was formed between the total score axis and the recurrence prediction axis.

**Figure 2:**
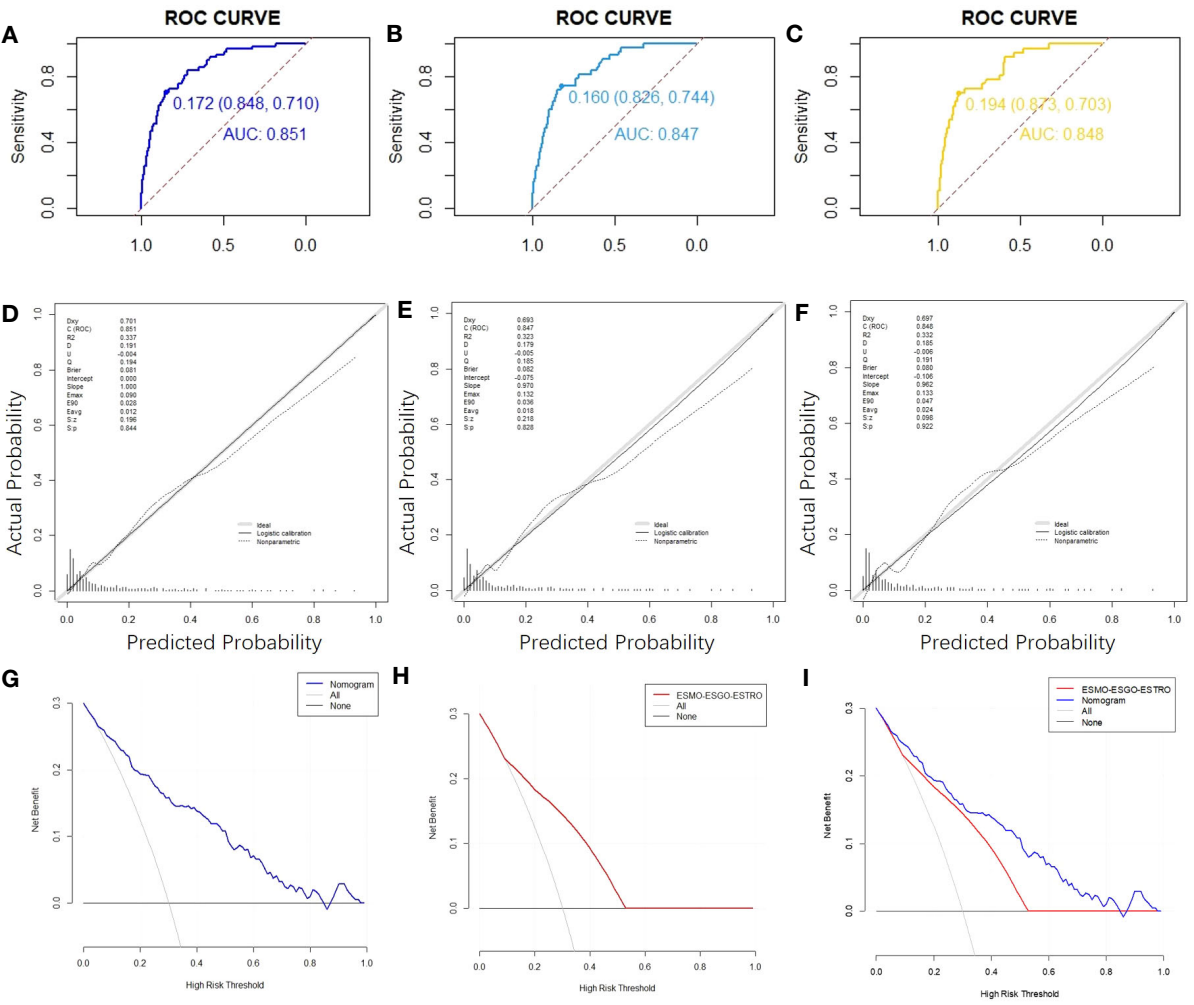
ROC curves,calibration charts and decision curve analysis of the recurrence prediction of patients with EC. **(A)** The ROC curve of the nomogram in the training cohort. **(B)** The nomogram’s ROC curves in validation cohort 1. **(C)** Nomogram ROC curves in validation cohort 2. **(D)** Calibration chart of the nomogram for the training cohort’s recurrence prediction of EC. **(E)** Nomogram calibration on EC recurrence prediction in validation cohort 1. **(F)** Calibration chart on EC recurrence prediction in validation cohort 2. **(G)** The nomogram’s DCA curve on training cohort’s EC recurrence prediction. **(H)** DCA curve of the ESMO-ESGO-ESTRO pattern for the recurrence prediction of EC in a training cohort. **(I)** Nomogram and ESMO-ESGO-ESTRO pattern comparison. DCA: Decision Curve Analysis.

We estimated the probability of EC recurrence by constructing two nomograms for EC recurrence based on total variables and eight screened covariates (**Figure 1**) that were independently associated with EC recurrence. In the nomogram model, **Figure 1** demonstrates how a nomogram may be used to forecast a patient’s chance of recurrence. Individual scores derived from a nomogram are used to compute total scores. The C-index value for the training cohort’s nomogram was 0.851 (95% confidence interval = 0.803-0.899) (**Figure 2A**), while the Hosmer-Lemeshow test indicated the existence of statistical significance on the two models’ difference (p = 0.850).

### Nomogram validation

The C-index value for the training cohort was 0.851 (95% confidence interval = 0.803-0.899); for validation cohort 1, it was 0.847 (95% confidence interval = 0.790-0.903); for validation cohort 2, it was 0.848 (95% confidence interval = 0.786-0.911) (**Figure 2B, C**). In all cohorts, the curves indicating nomogram calibration demonstrated significant correlation for anticipated and experimental recurrence probabilities (**Figures 2D-F**). In addition, the DCA curves indicated that the nomogram predicted recurrence in EC patients with a good level of discriminating (**Figures 2G-I**). Taken together, our constructed nomogram for predicting EC recurrence has considerable discriminative and calibration power.

### Nomogram clinical value against the ESMO-ESGO-ESTRO pattern

**Figure 3:**
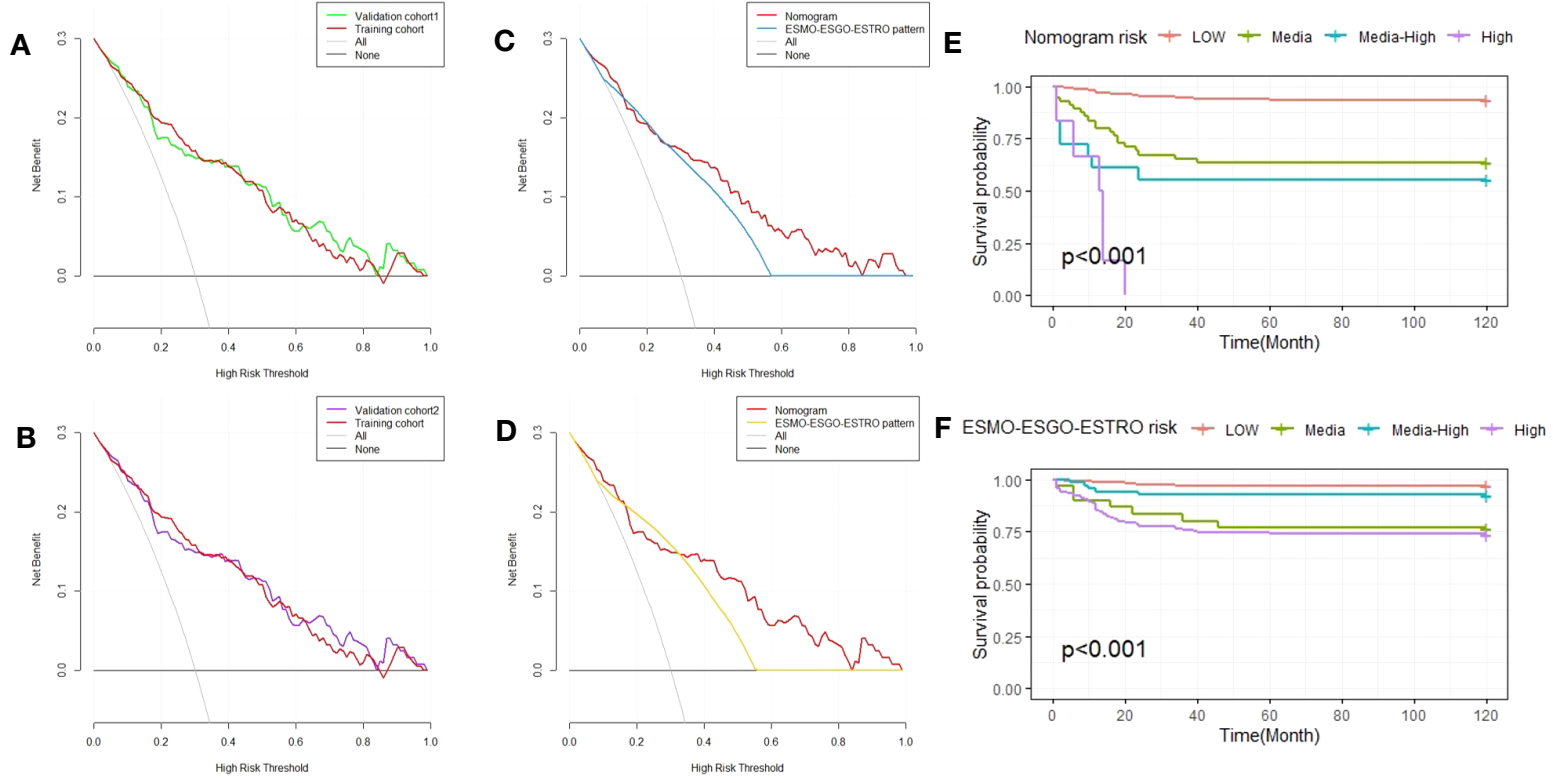
Decision curve analysis of the nomogram and ESMO-ESGO-ESTRO pattern for predicting EC recurrence, as well as Kaplan-Meier RFS curves for EC patients with varied risks. **(A)** A comparison of the validation 1 and training cohorts. **(B)** A comparison of the validation 2 and training cohorts. **(C)** Comparison of the nomogram and ESMO-ESGO-ESTRO pattern in validation cohort 1. **(D)** Comparison of the nomogram and the ESMO-ESGO-ESTRO pattern in validation cohort 2. **(E)** Nomogram-stratified Kaplan-Meier RFS curves for EC patients with varying risk levels in the training cohort. **(F)** Kaplan-Meier RFS curves of EC patients in the training group with differing risks according to classification using the ESMO-ESGO-ESTRO pattern. ESMO-ESGO-ESTRO refers to Medical Oncology, European Society of Gynaecological Oncology and European SocieTy for Radiotherapy & Oncology, while RFS denotes recurrence free survival.

E. Vizza (24) et al. judged the risk of recurrence according to ESMO-ESGO-ESTRO risk classes (low, intermediate, intermediate-high, and high-risk). We compare the accuracy of the nomograph and ESMO-ESGO-ESTRO risk models using the C-index, NRI, and IDI. The ESMO-ESGO-ESTRO risk class predicted recurrence with a C-index of 0.756 (95% CI = 0.701-0.812). Therefore, our newly constructed model was more accurate in predicting recurrence than the ESMO-ESGO-ESTRO risk class (P = 0.002). It was 0.406 (95% CI = 0.058-0.576) for NRI, while it was 0.11 (95%CI = 0.058-0.162, P < 0.05) for IDI. With validation from the validation cohort (**Table 3**), there was greater accuracy in the nomogram than ESMO-ESGO-ESTRO-based recurrence assessment of risk for predicting EC recurrence. The DCA curves indicated that the nomogram improved recurrence prediction in both the training and validation groups of EC patients, adding more net benefit than risk classes based on ESMO-ESGO-ESTRO (**Figures 3A-D**). Hence, the nomogram has a good discriminative potential for predicting the recurrence of EC in patients.

**Table 3:** NRI, IDI, and C-index of the nomogram and the ESMO-ESGO-ESTRO risk classes alone in recurrence prediction for EC patients.

	Training cohort			Validation cohort1			Validation cohort2		
Index	Estimate	95% CI	P value	Estimate	95% CI	P value	Estimate	95% CI	P value
NRI	0.406	0.058-0.576		0.347	0.008-0.590		0.340	0.047-0.643	
IDI	0.110	0.058-0.162	**<0.001**	0.070	0.007-0.132	**0.028**	0.103	0.030-0.175	**0.006**
C-index(ROC)									
The nomogram	0.851	0.803-0.899	**0.002**	0.847	0.790-0.903	0.088	0.848	0.786-0.911	**0.035**
The ESMO-ESGO-ESTRO risk classes	0.756	0.701-0.812		0.787	0.725-0.849		0.778	0.710-0.846	

NRI, net reclassification index; IDI, integrated discrimination improvement, C-index, concordance index.P values that have statistical significance (less than 0.05) were in bold.

Finally, risk stratification was performed according to the recurrence probability calculated from the nomogram. The LG-ESS patients were separated into three groups: those at low risk (probability<0.25), those at intermediate-risk (0.25≤probability<0.5), those at intermediate-high risk (0.5≤probability<0.75), and those at high-risk (probability≥0.75). The Kaplan-Meier RFS curve demonstrated substantial difference among the four risk categories (P < 0.001). The nomogram outperforms the ESMO-ESGO-ESTRO pattern in identifying groups at high risk (**Figures 3E, F**).

## Discussion

This research aims to develop a nomogram prediction model for endometrial cancer recurrence by gathering patient data. Using multivariate logistic regression and stepwise regression, eight variables were selected based on AIC minima and integrated into the nomogram design. In this study, all cases were used to build the endometrial cancer recurrence model, and the validation cohort was constructed by randomly selecting 70% and 60% of the patients. Previous studies have shown that age, histopathological type, myometrial invasion, FIGO stage, lymph node metastasis, lymphovascular invasion, and tumor grade are endometrial cancer recurrence risk factors (12, 25, 26). This study considers these influencing factors consistent with our model’s prediction results.

However, there is still some disagreement about the impact of surgical methods. For example, previous studies have found no statistically significant difference in the recurrence risk of early-stage endometrial cancer between laparoscopic and open surgery (27), while it is surgery in our nomogram model. Modality is deemed a risk factor for recurrence of endometrial cancer. This might be since our model now includes patients with all stages of endometrial cancer, not only the early stage.

Additionally, adjuvant treatment is a disputed subject. Patients with a high risk of recurrence following surgical resection and staging of endometrial cancer may be given adjuvant chemotherapy with carboplatin and paclitaxel (28). According to Nick Johnson et al. (29), adjuvant chemotherapy alone may greatly lower the chance of endometrial cancer recurrence, especially the probability of a first recurrence beyond the pelvis, which is consistent with the findings of our predictive model. Chemotherapy considerably reduced the nomogram’s AIC on our predictive model, indicating its value in predicting RFS in endometrial cancer. In contrast, radiotherapy had no significant effect on predicting the recurrence of endometrial cancer. It may be because radiotherapy patients are more advanced or have higher risk factors for recurrence. In addition, some patients received both radiotherapy and chemotherapy, which shows that excessive adjuvant therapy in endometrial cancer patients may not be beneficial.

Traditionally, the initial choice for predicting recurrence in patients with endometrial cancer has been to stratify the risk of recurrence of endometrial cancer based on the ESMO-ESGO-ESTRO consensus. Usually, this stratified model cannot accurately predict the recurrence of endometrial cancer. Such phenomena may be attributable to age, adjuvant treatment, and other characteristics not included in the ESMO-ESGO-ESTRO consensus recurrence risk categorization. Thus, we compared the variable-richer nomogram to the conventional ESMO-ESGO-ESTRO consensus conference-based recurrence risk categorization. Through C-index, NRI, IDI, and DCA curves, our nomogram predicts recurrence probability alongside a much greater clinical advantage and utilization compared to the ESMO-ESGO-ESTRO risk stratification system and can better identify high-risk groups.

The nomogram has shown possible clinical capacity. Data from all patients diagnosed with endometrial cancer in Nanjing Drum Tower Hospital between 2008 and 2018 were used, which represents different types of populations. We calculated the C-index or AUC, calibration curve, DCA curve, and others to evaluate the model, validating these results with a validation cohort. Taken together, our nomogram is a viable tool for determining the recurrence likelihood of endometrial cancer patients, and it may have therapeutic relevance for the postoperative monitoring and early diagnosis of disease recurrence in endometrial cancer patients. Despite the excellent performance of the nomogram, there are limits to this research. For example, some of the collected information is missing. In addition, clinical validation across multiple centers may be necessary for assessing nomograms’ external usefulness.

## Data availability statement

The data analyzed in this study is subject to the following licenses/restrictions: The data set is from a single medical center and has limitations. Requests to access these datasets should be directed to Mengdan Miao, mengdan_miao@njmu.edu.cn.

## Ethics statement

The studies involving humans were approved by The Medical Ethics Committee of Affiliated Drum Tower Hospital, Medical School, Nanjing University. The studies were conducted in accordance with the local legislation and institutional requirements. Written informed consent for participation was not required from the participants or the participants’ legal guardians/next of kin in accordance with the national legislation and institutional requirements.

## Author contributions

MM prepared the manuscript. All authors analyzed the data, read, and approved the final manuscript. HZ conceived and supervised the project. All authors contributed to the article and approved the submitted version.
